# Resveratrol and Arsenic Trioxide Act Synergistically to Kill Tumor Cells In Vitro and In Vivo

**DOI:** 10.1371/journal.pone.0098925

**Published:** 2014-06-05

**Authors:** Xiao-Yan Zhao, Shen Yang, You-Ran Chen, Pei-Chun Li, Meng-Meng Dou, Jie Zhang

**Affiliations:** 1 College of Pharmaceutical Sciences, Southwest University, Chongqing, China; 2 Department of Neurology, The Ninth People’s Hospital of Chongqing, Chongqing, China; Wayne State University School of Medicine, United States of America

## Abstract

**Background and Aims:**

Arsenic trioxide (As_2_O_3_), which used as an effective agent in the treatment of leukaemia and other solid tumors, is largely limited by its toxicity. QT prolongation, torsades de pointes and sudden heart death have been implicated in the cardiotoxicity of As_2_O_3_. The present study was designed to explore whether the combination of As_2_O_3_ and resveratrol could generate a more powerful anti-cancer effect both in vitro and in vivo.

**Materials and Methods:**

MTT assay was performed to assess the proliferation of Hela, MCF-7 and NB4 cells. Isobolographic analysis was used to evaluate combination index values from cell viability data. The apoptosis and the cellular reactive oxygen species (ROS) level were assessed by fluorescent microscopy and flow cytometry separately in vitro. The effect of As_2_O_3_, alone and in combination with resveratrol on Hela tumor growth in an orthotopic nude mouse model was also investigated. The tumor volume and the immunohistochemical analysis of CD31, CD34 and VEGF were determined.

**Results:**

Resveratrol dramatically enhanced the anti-cancer effect induced by As_2_O_3_ in vitro. In addition, isobolographic analysis further demonstrated that As_2_O_3_ and resveratrol generated a synergistic action. More apoptosis and ROS generation were observed in the combination treatment group. Similar synergistic effects were found in nude mice in vivo. The combination of As_2_O_3_ and resveratrol dramatically suppressed both tumor growth and angiogenesis in nude mice.

**Conclusions:**

Combining As_2_O_3_ with resveratrol would be a novel strategy to treat cancer in clinical practice.

## Introduction

Arsenic trioxide (As_2_O_3_) is clinically effective in treating acute promyelocytic leukemia [1]. The use of As_2_O_3_ to treat acute promyelocytic leukaemia began at the Harbin Medical University in the early of 1970s [2]. Related research also suggested that As_2_O_3_ showed substantial efficacy in a wide range of tumors including esophageal [3], cervical [4], lung [5] and liver carcinomas [6]. However, it was reported that the glutathione (GSH) system could generate arsenic detoxification in As_2_O_3_-resistant solid tumor cells. Consequently, some solid tumors, such as liver cancer and lung cancer, are less sensitive to As_2_O_3_ than acute promyelocytic leukemia. Furthermore, the clinical application of As_2_O_3_ was also limited by its toxicity in heart, liver, kidney and nerves system [7], especially the cardiac toxicity [8]. Combination therapy is a frequently used method in clinical practice to improve the therapeutic effect and reduce the toxicity of anticancer drugs [9]. Therefore, we attempt to find an agent which can enhance the anticancer effect of As_2_O_3_ and reduce its toxicity.

Resveratrol (trans-3, 4′, 5-trihydroxystilbene), a naturally-occurring polyphenolic compound, is highly enriched in a variety of food sources, such as grapes, peanuts and red wine [10]. Intriguingly, several pharmacological effects of resveratrol, including oestrogenic, cardiovascular protective, anti-inflammatory and antiplatelet effect, have been demonstrated [11]. In addition, some studies have shown that resveratrol has strong chemopreventive effects against the skin, breast, prostate and lung tumors [12]. The cancer preventive effects of resveratrol seem to be precise for it was shown to prevent tumor growth in animal model [13]. Furthermore, it was also reported that resveratrol can inhibit the growth of human cancer cells in vitro when it was used alone at rather high concentrations or in combination with other anticancer drugs [14]. On the other hand, our previous study also demonstrated that resveratrol significantly reduced the As_2_O_3_-induced cardiotoxicity in vitro and in vivo [15].

Based on these findings, we hypothesized that As_2_O_3_ combined with resveratrol would generate a more powerful anticancer effect than treatment with either agent alone. A series of studies were therefore performed in vitro and in vivo to investigate this hypothesis. The present data indicated that resveratrol significantly increased the anticancer effect induced by As_2_O_3_. Meanwhile, the mechanism of the synergistic effect of resveratrol and As_2_O_3_ has been preliminary studied.

## Materials and Methods

### Materials

DMSO, trypsin, resveratrol, penicillin, streptomycin, 3-[4, 5-dimethyl-2-thiazolyl]-2, 5-diphenyl-2-tetrazolium bromide (MTT) and acridine orange (AO) were purchased from sigma chemicals (St Louis, Mo, USA). The fetal bovine serum was obtained from Tianhang Biotechnology Company (Zhejiang, China). As_2_O_3_ was obtained from the Medical University Pharmaceutical Co., Ltd (Harbin, China). Annexin V-PI apoptosis assay kit was purchased from Roche Diagnostics Co., Ltd (Indianapolis, IN, USA).

### Cell Culture

The human cervical cancer Hela, human breast cancer MCF-7 and human APL NB4 cell lines were purchased from the Cell Bank of Chinese Academy of Sciences (Shanghai, China). All cell lines were cultured in Dulbecco’s Modified Eagle Medium supplemented with 10% fetal bovine serum, 100 U/ml penicillin and 100 µg/ml streptomycin at 37°C in 5% CO_2._ Cells were passaged and subcultured to 90% confluence with 0.25% trypsin (w/v) every 2–3 days.

### Cell Proliferation Assay

Hela, MCF-7 and NB4 cells were collected with trypsin and re-suspended in a final density of 5×10^4^ cells per ml, and then seeded in 96-well plates. To evaluate the synergistic effects of resveratrol and As_2_O_3_, Hela cells were treated with resveratrol (0.1, 1, 10, 100, 200 µM), As_2_O_3_ (0.1, 1, 10, 100, 200 µM), and resveratrol combined with As_2_O_3_ (17+1, 85+5, 170+10, 340+20, 680+40 µM) for 48 h. MCF-7 cells were treated with resveratrol (0.1, 1, 10, 100, 200 µM), As_2_O_3_ (0.1, 1, 10, 100, 200 µM), resveratrol+As_2_O_3_ (6+0.5, 12+1, 18+1.5, 36+3, 72+6 µM) for 48 h. And NB4 cells were treated with resveratrol (0.1, 1, 10, 100, 200 µM), As_2_O_3_ (0.1, 1, 10, 100 µM), resveratrol+As_2_O_3_ (6+0.5, 12+1, 36+3, 72+6, 120+10 µM) for 48 h. Then cell viability was assessed by the MTT assay as described previously [16]. Briefly, 10 µl of the MTT (5 mg/ml) reagent was added to each well and incubated for 4 h. The cell supernatants were removed and DMSO was added to dissolve the formazan crystals. The absorbance was measured with a BIO RAD microplate reader (model 630, USA) at 490 nm. The relative cell viability was expressed as the percentage of control well (not treated with drugs).

### Analysis of Drug Synergism

The combination index (CI) was used to evaluate whether the two drugs have a synergistic, antagonistic or additive effect, as reported previously [17]. The CI is calculated as the following formula: CI = (D)_1_/(D_x_)_1_+ (D)_2_/(D_x_)_2_, in which (D)_1_ represent the concentration of compound achieving a particular effect in the combination; the (D_x_)_1_ is the concentration of the same drug that will cause the identical effect by itself; (D)_2_ is the concentration of the other drug which will achieve a particular effect in the combination; and (D_x_)_2_ is the other drug that will generate the same level of effect by itself. CI>1 indicates antagonism, CI = 1 represents additivity, and CI<1 shows synergy [16].

### Fluorescent Microscopy Measurements

Acridine orange (AO) staining was used for detecting the apoptotic or necrotic cells [18]. In the present study, Hela and MCF-7 cells were seeded in 6 well plates at a density of 80,000 cells per well. Cells were treated with different concentrations of resveratrol and As_2_O_3_ (the control group only incubated with culture medium) for 48 h. Then, 10 µl of prepared AO working solution (100 µg/ml in PBS) was added to each well. The cells were immediately examined with a fluorescence microscope (Olympus U-RFLT50, Tokyo, Japan). Morphologically apoptotic and necrotic cells were counted in 10 visual fields of 5 different areas.

### Cell Apoptosis Analysis by Flow Cytometry

To investigate the cell apoptosis, Annexin V-FITC/PI staining was performed. Hela and NB4 cells were exposed to different treatments (medium, 50 µM resveratrol, 3 µM As_2_O_3_, 50 µM resveratrol +3 µM As_2_O_3_) for 48 h. The floating and trypsinized adherent cells were then collected and detected as described previously [19], [20]. Cell apoptosis was detected using Annexin V-FITC/PI Apoptosis Detection Kit according to the manufacturer’s instructions with a FACSCalibur machine (Becton Dickinson, San Jose, CA, USA).

### Measurement of ROS Production

DCFH-DA fluorescent labeling was used to measure intracellular production of the reactive oxygen species (ROS) in Hela cells. For the procedure, Hela cells were exposed to resveratrol (50 µM), As_2_O_3_ (3 µM), or resveratrol+As_2_O_3_ (50 µM +3 µM) for 48 h. Then the cell supernatants were removed and 2′, 7′-dichlorofluorescin-diacetate (DCFH-DA) was added into each group. After incubation with DCFH-DA for 30 min at 37°C, the cells were washed twice with PBS and maintained in 1 ml serum-free medium. The fluorescence images were captured by a fluorescence microscope (OLYMPUS U-RFLT50, Japan), under ×200 magnification with the filter which excitation at 470–490 nm and emission at 510–550 nm.

### Animal Treatment

35 male BALB/c nude mice were purchased from Animal Center, Chongqing Medical University, Chongqing, China. The mice were provided food and water freely on a 12∶12 h day-night cycle with room temperature maintained at around 26°C. 2×10^6^ Hela cells were injected subcutaneously into the armpit of the mice. Body weight of the mice was recorded every day and the tumor growth was monitored every three days. When the average tumor volume was reached 100 mm^3^, tumor-bearing mice were randomly divided into four groups, respectively treated with vector, 16.5 mg/kg/d resveratrol, 5 mg/kg/d As_2_O_3_, or the combination of resveratrol and As_2_O_3_ for 2 weeks. The tumor growth and body weight of the mice were monitored every day. The volume of the tumor was calculated from the formula V = 1/2 (A×B^2^), where A was the longest diameter and B was perpendicular diameter measured by calipers.

### Inhibition of Tumor Growth

When treatment was finished, the mice were sacrificed and the tumors were excised for analysis. Excised tumors were weighted and then fixed with formalin. 5 µm tissue sections were stained with haematoxylin and eosin. Morphological examination was conducted under a light microscope.

### Microvessel Density was Assessed by Immunohistochemical Staining

Tumor vascularity was determined by staining tumor sections with antibodies to CD31, CD34 and VEGF. Tumor tissues were fixed in 10% formalin solution for paraffin embedding. The paraffin-embedded sections of tumors were cut at 4 µm and endogenous peroxidase activity in the sections was blocked by treating with 3% H_2_O_2_ for 15 min. Antigen retrieval was performed by boiling the sections for 10 min in citrate buffer (pH = 6.0) and cooling them naturally, followed by incubation in the blocking buffer (PBS +10% NGS +0.1% Triton X-100) for 1 h. The sections were subsequently incubated overnight at 4°C with optimal dilutions of anti-CD31, anti-CD34 and anti-VEGF antibodies. The Peroxidase Substrate DAB kit was used for detection and visualization of staining following the manufacturer’s instructions. Finally, images were taken using an inverted Olympus fluorescence microscope.

### Statistical Analysis

All statistical analysis was performed using PASW Statistical software (version 18.0 for Windows). Values were presented as the mean ± SD. Statistical comparisions were performed by one-way ANOVA. Tukey’s post hoc test was used for multiple group comparisons and Student’s t-test was used for single comparisons. *P*<0.05 was considered to be statistically significant.

### Ethics Statement

This study was carried out in strict accordance with the recommendations in the Guide for the Care and Use of Laboratory Animals of the National Institutes of Health. All animal procedures were approved by the Ethical Committee for Animal Experiments of Southwest University (Permit Number: SYXK 2009-0002). All efforts were made to minimize suffering.

## Results

### Resveratrol Enhanced the Antiproliferative Effect Induced by As_2_O_3_ in Different Cancer Cell Lines

**Figure 1:**
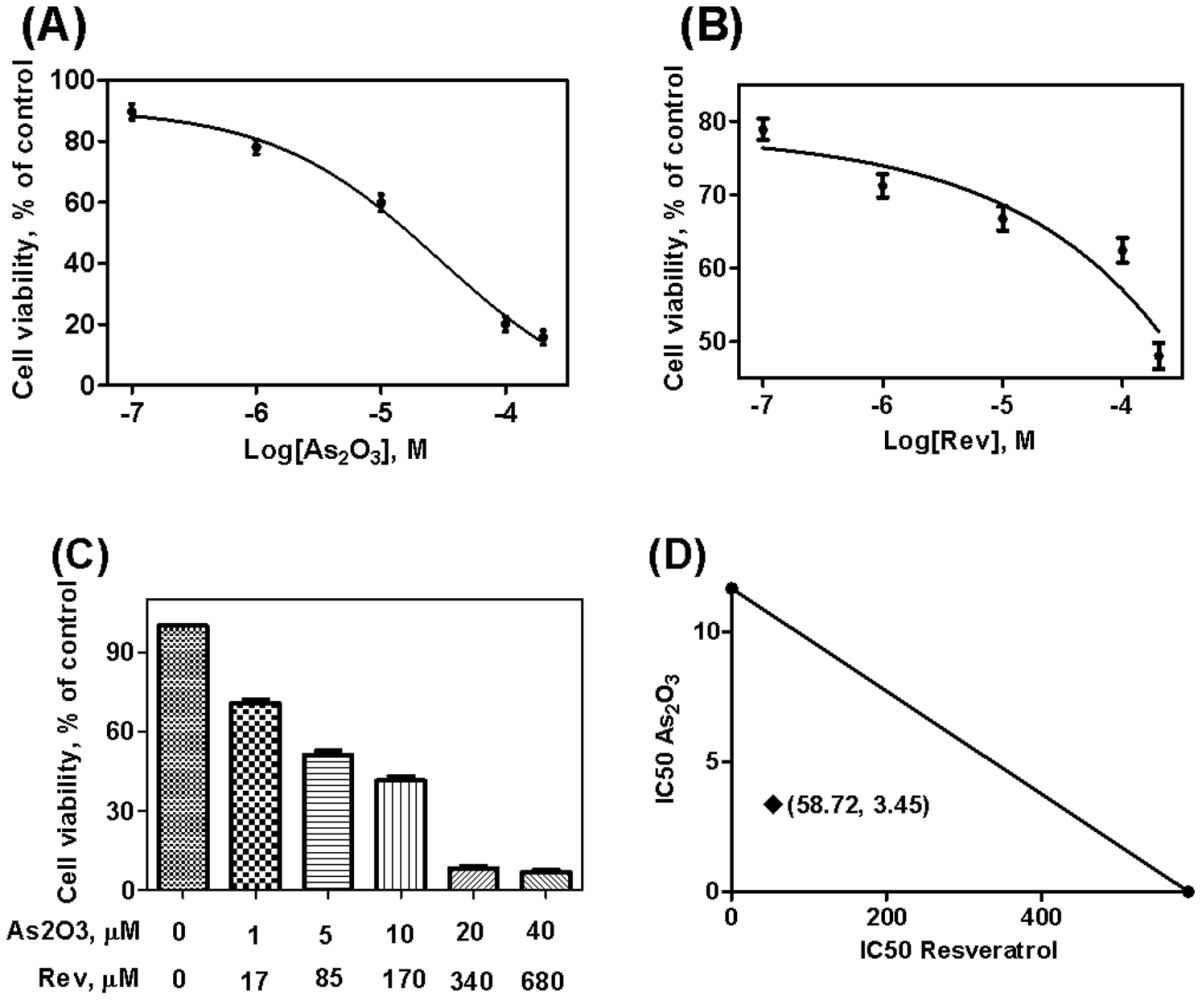
Effects of resveratrol on the cell proliferation in As_2_O_3_-treated Hela cells. Hela cells were incubated with different concentrations of resveratrol (A), As_2_O_3_ (B) and the combination of resveratrol and As_2_O_3_ (C) for 48 h, the cell proliferation was then evaluated by MTT assay. Values represent Mean±SD of three independent experiments. Isobologram illustration in Hela cells for the combination of resveratrol and As_2_O_3_, the point (58.72, 3.45) is the IC50 of combined resveratrol and As_2_O_3_ (D).

**Figure 2:**
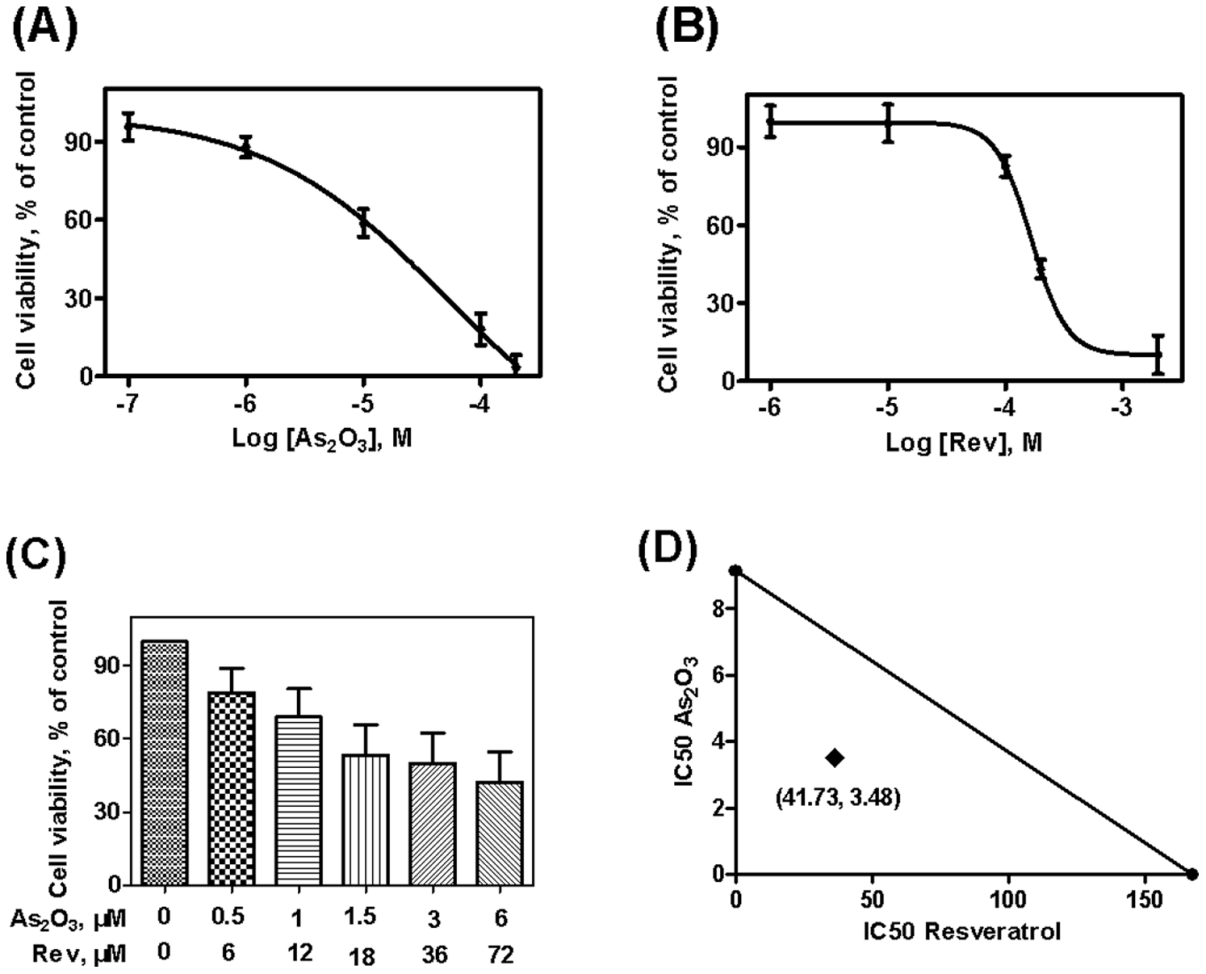
Effects of resveratrol on the cell proliferation in As_2_O_3_-treated MCF-7 cells. MCF-7 cells were incubated with different concentrations of resveratrol (A), As_2_O_3_ (B) and the combination of resveratrol and As_2_O_3_ (C) for 48 h, the cell proliferation was then evaluated by MTT assay. Values represent Mean±SD of three independent experiments. Isobologram illustration in MCF-7 cells for the combination of resveratrol and As_2_O_3_, the point (41.73, 3.48) is the IC50 of combined resveratrol and As_2_O_3_ (D).

**Figure 3:**
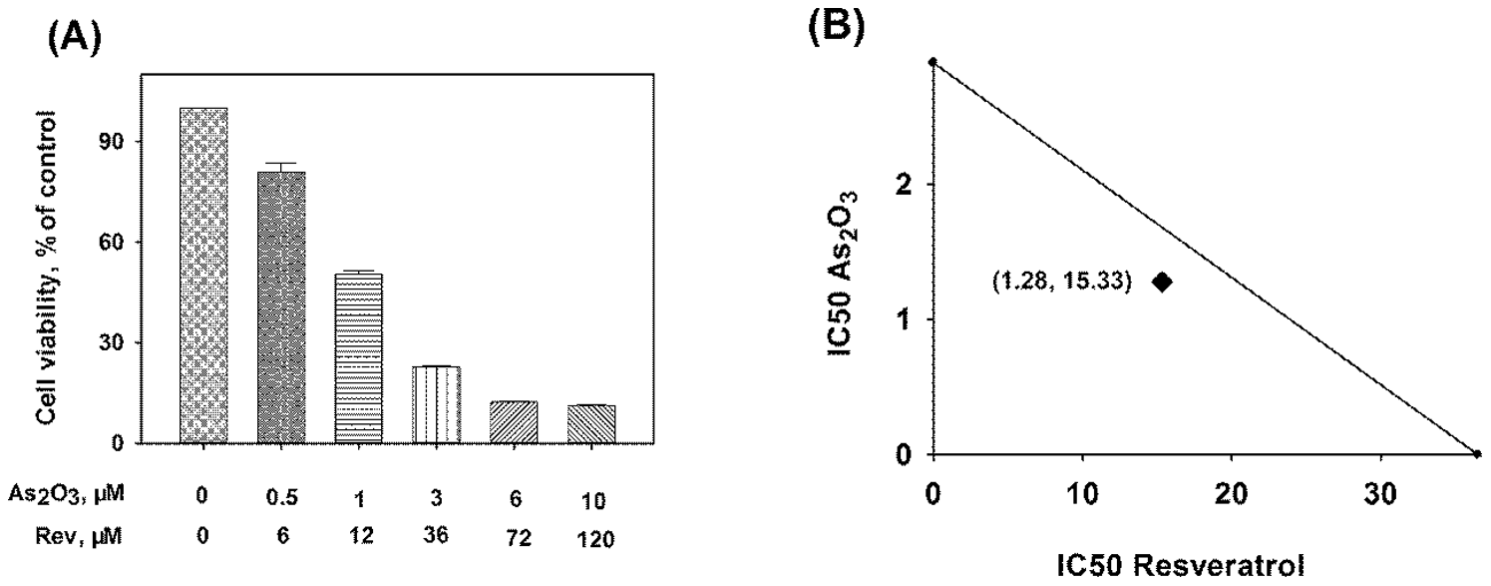
Effects of resveratrol on the cell proliferation in As_2_O_3_-treated NB4 cells. NB4 cells were incubated with different concentrations of the combination of resveratrol and As_2_O_3_ for 48 h, the cell proliferation was then evaluated by MTT assay (A). Values represent Mean±SD of three independent experiments. Isobologram illustration in NB4 cells for the combination of resveratrol and As_2_O_3_, the point (1.28, 15.33) is the IC50 of combined resveratrol and As_2_O_3_ (B).

To explore the effects of resveratrol and As_2_O_3_ on the viability of Hela, MCF-7 and NB4 cells, MTT assay was performed. The cell inhibition rate in Hela, MCF-7 and NB4 cells exposed to different concentrations of resveratrol, As_2_O_3_, or the combination of resveratrol and As_2_O_3_ are shown in Fig. 1, Fig. 2 and Fig. 3. The concentration-depended cell inhibition rate after treatment with resveratrol and As_2_O_3_ was found in the three cell lines. All the cell lines were sensitive to resveratrol and As_2_O_3_ treatment. Moreover, combination of resveratrol and As_2_O_3_ produced significantly higher inhibition rates compared with single-agent treatment at the same concentration in respective cells.

### Synergistic Action of the Combination of Resveratrol and As_2_O_3_

Drug combination analysis with isobolograms was carried out to investigate whether the combination of resveratrol with As_2_O_3_ could have a synergistic effect. As shown in Fig. 1D, Fig. 2D and Fig. 3B, Y-axis represented the IC50 of resveratrol and X-axis represented the IC50 of As_2_O_3_. The points represented the IC50 of resveratrol and As_2_O_3_ in combination group. Then the IC50 of resveratrol and As_2_O_3_ were connected with a straight line. The points were all below the additivity lines, which suggested that combination of resveratrol with As_2_O_3_ had a synergistic anticancer effect in Hela, MCF-7 and NB4 cells.

### Combined Treatment with As_2_O_3_ and Resveratrol Induced More Apoptosis in vitro

**Figure 4:**
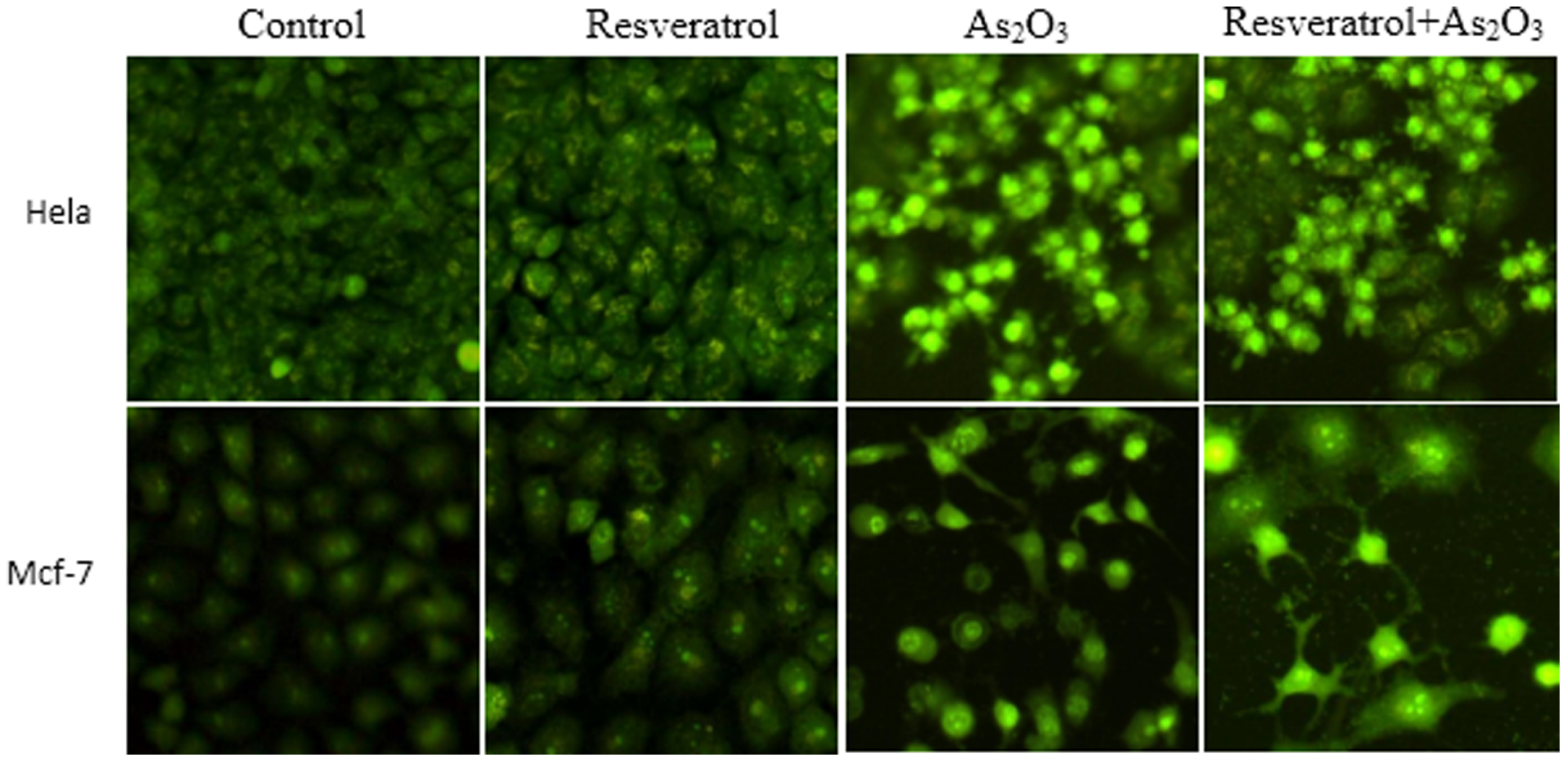
Apoptosis in Hela and MCF-7 cells were evaluated by fluorescent microscopy measurements. Hela and MCF-7 cells were treated with culture medium, resveratrol, As_2_O_3_, and combination of resveratrol and As_2_O_3_ for 48 h, then stained with AO and examined with a fluorescent microscope (×200).

**Figure 5:**
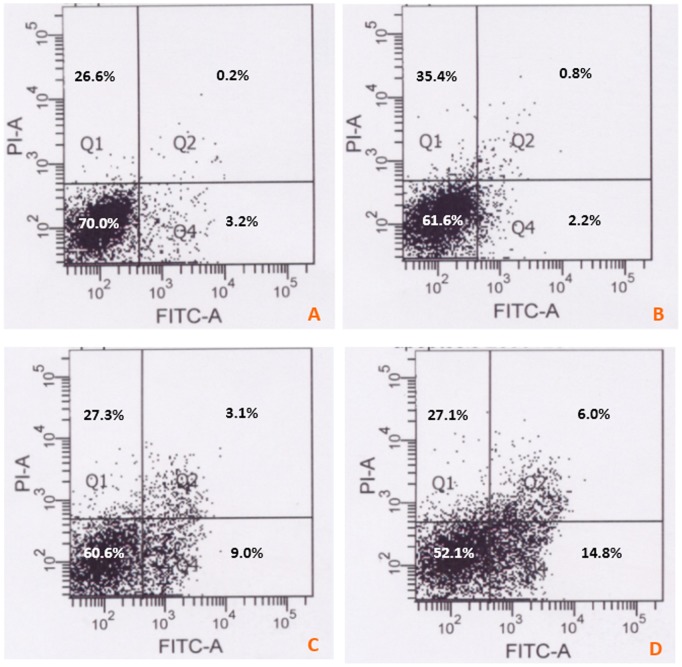
Apoptosis in NB4 cells were quantitated by flow cytometry. NB4 cells were treated with culture medium (A); 50 µM resveratrol (B); 3 µM As_2_O_3_ (C) and combination of resveratrol and As_2_O_3_ (D) for 48 h, then PI-FITC was carried out to investigate the apoptosis rate.

To evaluate the apoptosis induced by combined treatment of As_2_O_3_ and resveratrol, fluorescent microscopy measurement and flow cytometry analysis were carried out. The results of fluorescent microscopy measurements (AO staining) were shown in Fig. 4. Annexin V-FITC/PI assays in NB4 cells were shown in Fig. 5. These data suggested that combination treatment of As_2_O_3_ with resveratrol induced more apoptosis in cancer cells than As_2_O_3_ alone.

### Combined Treatment with As_2_O_3_ and Resveratrol Generated More ROS in Hela Cells

**Figure 6:**
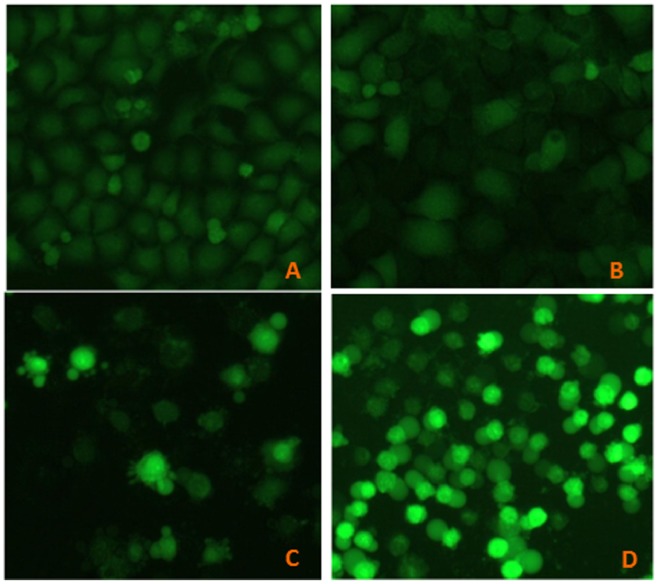
Change of the intracellular ROS production in Hela cells. Hela cells were treated with culture medium (A); 50 µM resveratrol (B); 3 µM As_2_O_3_ (C) and resveratrol combined with As_2_O_3_ (D) for 48 h, then the intracellular ROS assay was performed, and captured under a fluorescence microscope (×200).

DCFH-DA fluorescent labeling was used to evaluate ROS in Hela cells treated with As_2_O_3_, resveratrol or the combination. The data was shown in Fig. 6, which demonstrated that combination of As_2_O_3_ and resveratrol could generate more ROS in Hela cells.

### Tumor Growth was Inhibited by Combination of As_2_O_3_ and Resveratrol in the Xenografts Model in Nude Mice

**Figure 7:**
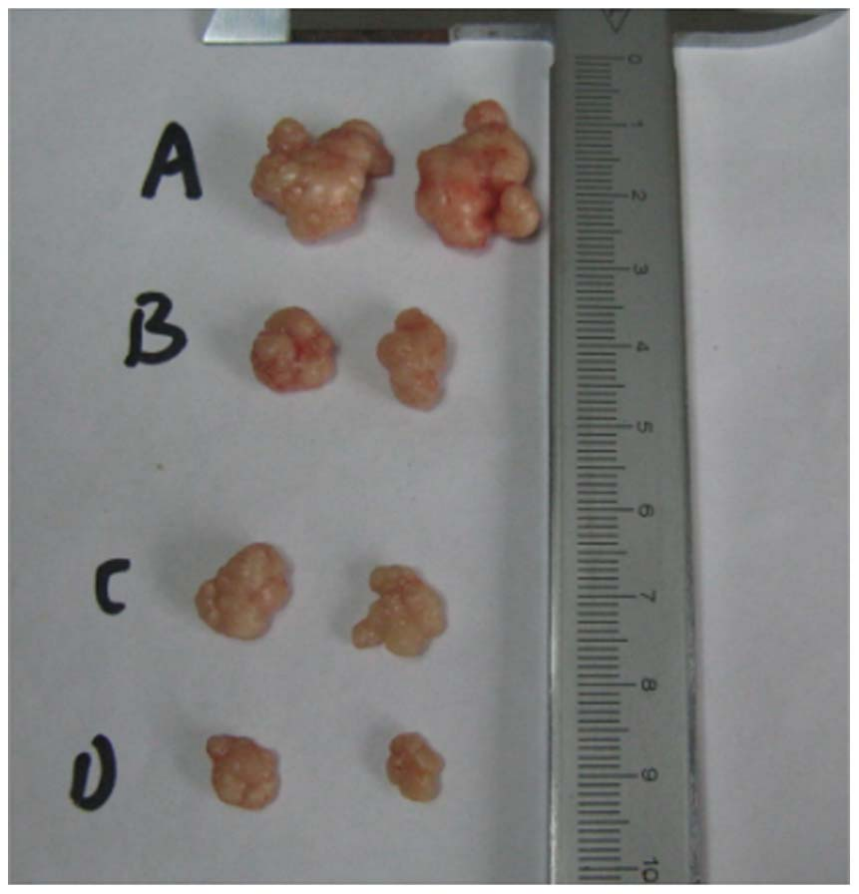
The tumor weight and volume in nude mice. After 2 weeks treatment with vector (A), 16.5 mg/kg/d resveratrol (B), 5 mg/kg/d As_2_O_3_ (C), or combination of resveratrol and As_2_O_3_ (D), the tumors were excised and weighted.

**Figure 8:**
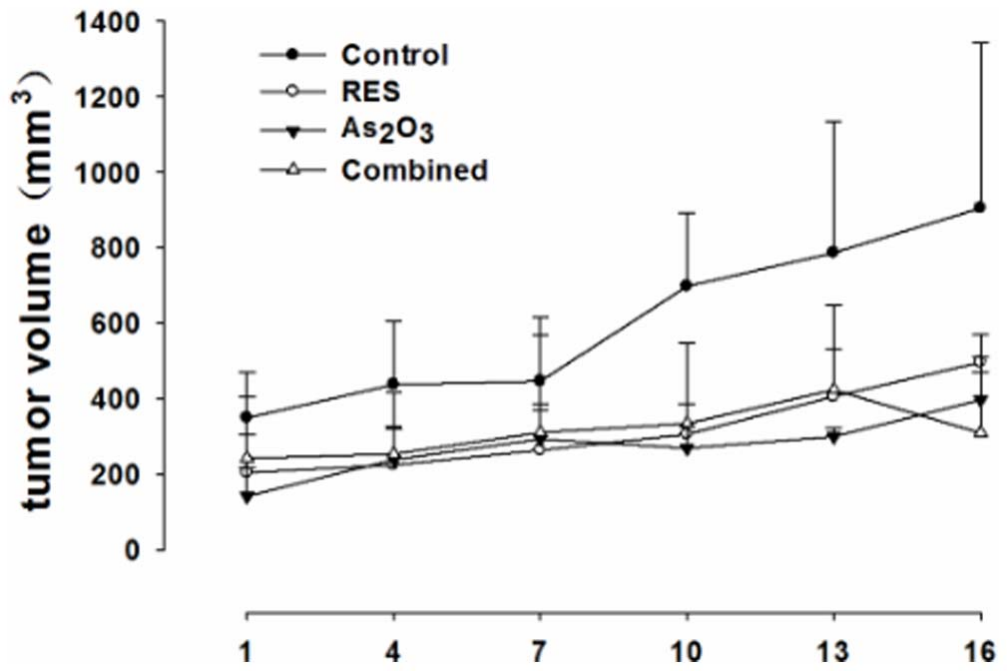
The relative tumor volume change (compared with the tumor volume before drugs treatment) of nude mice. 35 nude mice were randomly divided into 4 groups and treated with vector, 16.5/kg/d resveratrol, 5 mg/kg/d As_2_O_3_, or combination of resveratrol and As_2_O_3_ for 2 weeks respectively. The tumor volume was monitored every day, and the tumor volume was calculated from the formula V = 1/2 (A×B^2^), where A was the longest diameter and B was perpendicular diameter measured by calipers.

No difference was found in body weight of nude mice treated with As_2_O_3_, resveratrol, or As_2_O_3_ plus resveratrol. In contrast, a significant reduction in tumor volume was evident after treatment with As_2_O_3_ or resveratrol alone. Moreover the reduction was further enhanced when As_2_O_3_ and resveratrol were used in combination (*P*<0.05). (Fig. 7, Fig. 8).

### More Necrosis in the Xenograft Model was Occurred by Combined Treatment of Resveratrol and As_2_O_3_

**Figure 9:**
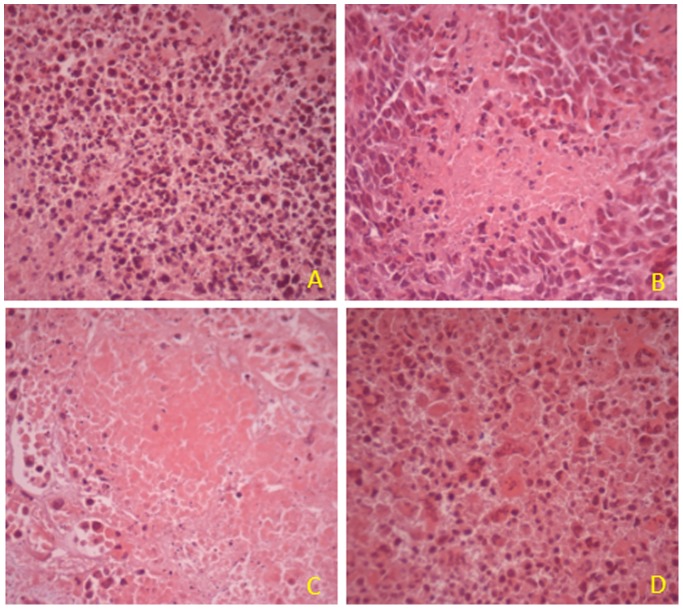
HE staining of the tissue slices from xenograft tumors. The excised tumors of mice which treated with vector (A), 16.5 mg/kg/d resveratrol (B), 5 mg/kg/d As_2_O_3_ (C), or combination of resveratrol and As_2_O_3_ (D), were fixed with formalin and sliced, then HE staining were performed.

To evaluate whether the combination of resveratrol and As_2_O_3_ induced more apoptosis or necrosis in the xenografts of Hela cell, HE staining was performed. As shown in Fig. 9, necrosis was found in resveratrol or As_2_O_3_-treated group. However, more necrosis was observed in the combination group than the other groups.

### Immunohistochemical Staining for the Tumor Vascularity (CD31, CD34 and VEGF)

**Figure 10:**
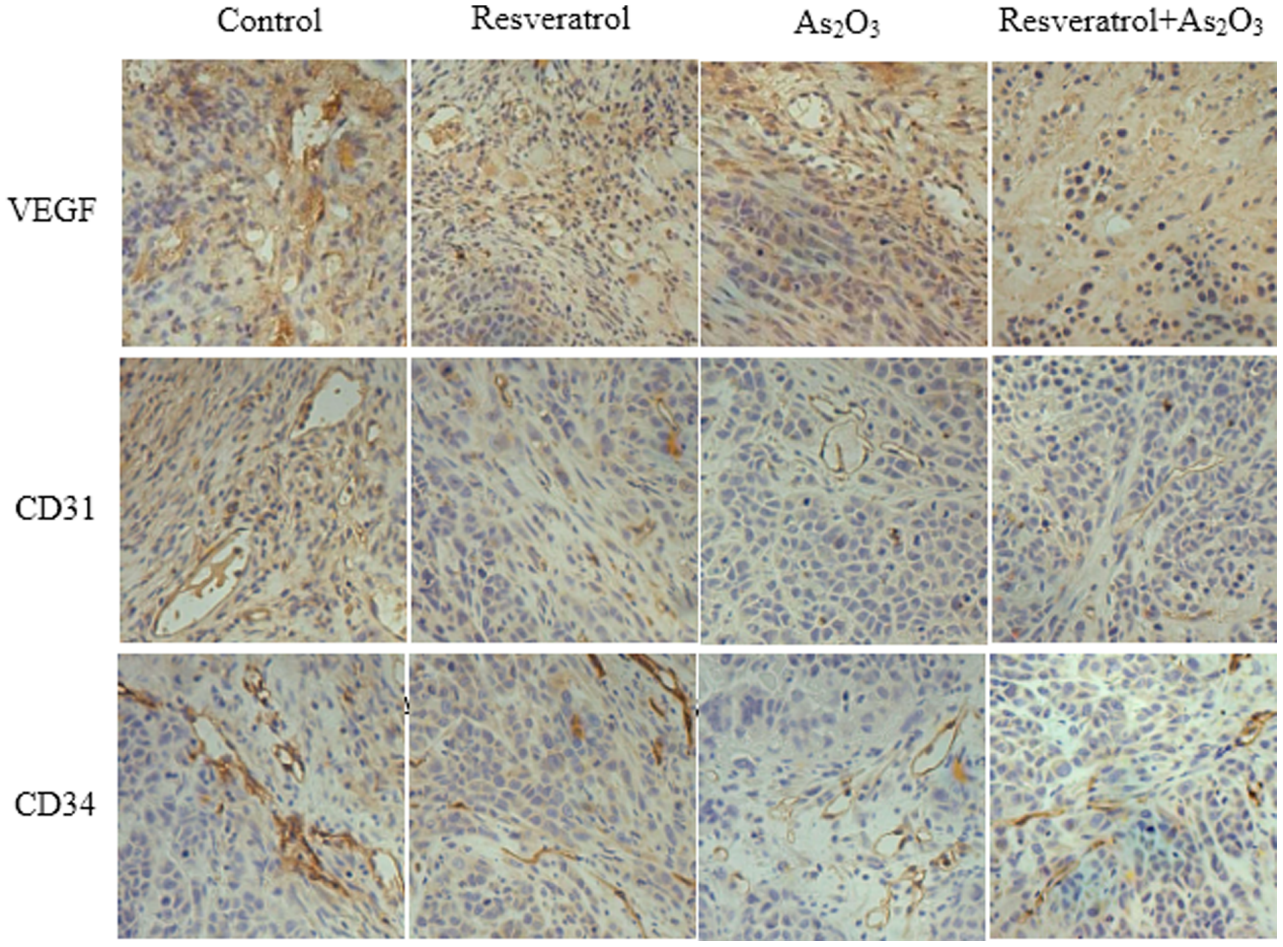
The tumor vascularity was detected by the immunofluorescent staining of CD31, CD34 and VEGF. The xenograft tumors treated with vector, 16.5/kg/d resveratrol, 5 mg/kg/d As_2_O_3_, or combination of resveratrol and As_2_O_3_ were stained for CD31, CD34 and VEGF, and then captured under a fluorescence microscope.

Immunohistochemical staining of CD31, CD34 and VEGF was carried out to detect the tumor vascularity. It was shown in Fig. 10. The immunohistochemical expression of CD31 and CD34 were used to demonstrate the presence of endothelial cells in xenografts tissue sections, and VEGF was established as a major factor in regulating angiogenesis. The expression of CD31, CD34 and VEGF in the combination group was lower than other groups, which indicated that the combination of resveratrol and As_2_O_3_ achieved a stronger inhibition of the tumor vascularity.

## Discussion

In our present study, the results showed that resveratrol enhanced the anti-cancer effect of As_2_O_3_ in vitro and in vivo. The cell proliferation assay supported that resveratrol significantly increased the anti-proliferation of As_2_O_3_ in a dose dependent manner. In addition, the isobologram analysis demonstrated that combination of resveratrol with As_2_O_3_ had a synergistic effect. The results of fluorescent microscopy measurements and flow cytometry also demonstrated that resveratrol increased the apoptosis induced by As_2_O_3_ in vitro. The DCFH-DA fluorescent labeling assay suggested that resveratrol strengthened As_2_O_3_-induced anticancer effect through increasing the ROS level in cancer cells. The data in vivo further confirmed that resveratrol plus As_2_O_3_ notably suppressed both tumor growth and angiogenesis in the xenografts model.

It has been reported that resveratrol inhibits the proliferation of a wide variety of tumor cell lines [21]. Resveratrol as a prevention agent of cancer was reported previously [22]. Recent studies have shown that As_2_O_3_ induced apoptosis in cancer cells [23]. Meanwhile, our previous study showed that resveratrol can attenuate As_2_O_3_-induced cardiotoxicity in vitro and in vivo [15]. Therefore, we attempted to testify if the combination of As_2_O_3_ with resveratrol in cancer therapy has better anticancer effect and less toxicity than alone.

To approve the hypothesis, a serious of experiments had been performed. Firstly, the cell proliferation experiment indicated that resveratrol significantly increases the anticancer effect induced by As_2_O_3_ in Hela, MCF-7 and NB4 cells. Secondly, the isobolograms further demonstrated that As_2_O_3_ and resveratrol had the synergistic effect.

Current evidence suggests that resveratrol enhances the antileukemic properties of As_2_O_3_ [24]. Thus we investigated whether the combination of As_2_O_3_ and resveratrol has stronger antitumor effect in various cancer cell lines compared with As_2_O_3_ or resveratrol alone. The fluorescent microscopy measurements and flow cytometry assay indicated that resveratrol can significantly enhance cell apoptosis induced by As_2_O_3_. The study also suggested that resveratrol and As_2_O_3_ have a significant synergic anticancer effect in a xenografts model. Combination of resveratrol and As_2_O_3_ significantly reduced the tumor volume in nude mice. Expression of CD31, CD34 and VEGF also proved that the combination of resveratrol and As_2_O_3_ suppressed the microvascular of xenograft tumor. To my knowledge, inhibition of tumor vascularity was thought as a novel mechanism of chemotherapy [25]–[27].

The cardiotoxicity of As_2_O_3_ was reported to be associated with As_2_O_3_-induced ROS and intracellular Ca^2+^ overload [28]. As_2_O_3_-induced Long QT Syndrome (LQTS), a serious heart disease, was related to intracellular Ca^2+^ overload induced by As_2_O_3_. In addition, resveratrol can significantly decrease the intracellular Ca^2+^ [29]. Resveratrol can attenuate the cardiotoxicity induced by chemotherapy, such as QT prolongation, torsades de pointes and sudden cardiac death [30], [31]. Consequently, combination of resveratrol and As_2_O_3_ could not only increase the anticancer effect, but also decrease the cardiotoxicity induced by As_2_O_3_. Similarly, As_2_O_3_ can also cause damage on liver, kidney and nervous system [32], [33], so the combination therapy of resveratrol and As_2_O_3_ may reduce these toxicities. It is a novel strategy to prevent As_2_O_3_-induced cardiotoxicity in cancer patients.

It is reported that resveratrol possessed a potent anticancer effect in vitro when the concentration of resveratrol was high (usually higher than 50 µM) [34]. Our previous study had reported that resveratrol protected against the cardiotoxicity induced by As_2_O_3_ (lower than 10 µM) [15]. So, controlling the distributed of resveratrol is a crucial point in the combination treatment of resveratrol and As_2_O_3_. The target is that making more resveratrol accumulated in the cancerous tissues and less resveratrol in the normal tissues. Maybe the targeted drug delivery system could be used in the administration of resveratrol.

In conclusion, the present study suggested that resveratrol and As_2_O_3_ had a synergistic antitumor effect in vitro and vivo. Resveratrol effectively increased the apoptosis and intracellular ROS induced by As_2_O_3_. Combination of resveratrol and As_2_O_3_ can significantly reduce tumor volume in nude mice by both antitumor and antiangiogenic effects. Combining resveratrol with As_2_O_3_ is a hopeful strategy in clinical therapy for cancer. Further investigation will be performed to explore the molecular mechanism of the synergistic effects of resveratrol plus As_2_O_3_.
